# Lung microbiota across age and disease stage in cystic fibrosis

**DOI:** 10.1038/srep10241

**Published:** 2015-05-14

**Authors:** Bryan Coburn, Pauline W. Wang, Julio Diaz Caballero, Shawn T. Clark, Vijaya Brahma, Sylva Donaldson, Yu Zhang, Anu Surendra, Yunchen Gong, D. Elizabeth Tullis, Yvonne C. W. Yau, Valerie J. Waters, David M. Hwang, David S. Guttman

**Affiliations:** 1 Department of Cell and Systems Biology, University of Toronto, Toronto, Ontario, Canada; 2 Centre for the Analysis of Genome Evolution and Function, University of Toronto, Toronto, Ontario, Canada; 3 Latner Thoracic Surgery Laboratories, University Health Network, University of Toronto, Toronto, Ontario, Canada; 4 Department of Pathology, University Health Network, University of Toronto, Toronto, Ontario, Canada; 5 Adult Cystic Fibrosis Clinic, St. Michael’s Hospital, Toronto, Ontario, Canada; 6 Department of Pediatric Laboratory Medicine, Microbiology, The Hospital for Sick Children, Toronto, Ontario, Canada; 7 Department of Laboratory Medicine and Pathobiology, University of Toronto, Ontario, Canada; 8 Division of Infectious Diseases, Department of Pediatrics, Hospital for Sick Children, University of Toronto, Toronto, Ontario, Canada

## Abstract

Understanding the significance of bacterial species that colonize and persist in cystic fibrosis (CF) airways requires a detailed examination of bacterial community structure across a broad range of age and disease stage. We used 16S ribosomal RNA sequencing to characterize the lung microbiota in 269 CF patients spanning a 60 year age range, including 76 pediatric samples from patients of age 4–17, and a broad cross-section of disease status to identify features of bacterial community structure and their relationship to disease stage and age. The CF lung microbiota shows significant inter-individual variability in community structure, composition and diversity. The core microbiota consists of five genera - *Streptococcus, Prevotella, Rothia, Veillonella* and *Actinomyces*. CF-associated pathogens such as *Pseudomonas, Burkholderia, Stenotrophomonas* and *Achromobacter* are less prevalent than core genera, but have a strong tendency to dominate the bacterial community when present. Community diversity and lung function are greatest in patients less than 10 years of age and lower in older age groups, plateauing at approximately age 25. Lower community diversity correlates with worse lung function in a multivariate regression model. Infection by *Pseudomonas* correlates with age-associated trends in community diversity and lung function.

Cystic fibrosis (CF) is characterized by recurrent airway infection, inflammation and progressive decline in lung function. Infection with key organisms such as *Pseudomonas aeruginosa* and *Burkholderia cepacia* complex (Bcc) has been associated with the frequent exacerbations of airway dysfunction and progressive functional decline that are the hallmarks of the disease^1^,^2^,^3^.

Over the last decade, cross-sectional and longitudinal studies of bacterial taxa using culture-independent microbial detection methods such as terminal restriction fragment length polymorphism (T-RFLP) and 16S rRNA gene sequencing, have identified polymicrobial communities in the airways of CF patients that exceed the complexity captured by traditional culture^4^,^5^,^6^,^7^,^8^,^9^,^10^,^11^,^12^,^13^. Significant heterogeneity in bacterial community composition has been demonstrated within groups of clinically similar patients^5^,^6^,^7^. Although the abundance of individual taxa (such as *Gemella* spp. and the *Streptococcus anginosus* group), ecological diversity, and community stability are potentially associated with disease status^8^,^10^,^14^, no single static or dynamic metric or bacterial taxon has emerged that consistently explains the heterogeneity of disease within otherwise similar hosts.

Culture-based methods have demonstrated the clinical significance of infection with pathogens such as *P. aeruginosa*. Molecular methods have demonstrated that not only their presence or absence, but also their relative abundance, dominance over other taxa, and temporal stability are potentially important determinants of ecological and clinical change in lung disease^10^,^15^. While studies of the CF microbiome to date have largely focused on adult patients, we expand this base of knowledge by including 76 pediatric patients less than 18 years old (28% of the total sample). This early time period is particularly interesting from the perspective of CF microbiology since community composition is more dynamic in pediatric patients than in adults^16^,^17^. Here, we report a cross-sectional survey of the bacterial community in sputum samples from 269 CF patients spanning a 60-year age range and a wide range of lung function.

## Results

### Clinical and microbiological characteristics of patients

During the study period, 76 pediatric (<18 years old) and 193 adult CF patients provided at least one expectorated sputum sample for analysis. Complete clinical data was available for 73 pediatric and 191 adult patients. The characteristics of these patients are reported in Table 1. Pancreatic insufficiency was more common in pediatric patients (*P* < 0.001). A larger proportion of adult patients had intermediate (FEV_1_ < 70% predicted) or advanced (FEV_1_ < 40% predicted) disease (68.6% vs 44.3% of pediatric patients, *P* = 0.001) and the median FEV_1_ was lower in the adult cohort, reflecting the progression of disease with increasing age (*P* < 0.001). Adult patients were more likely than pediatric patients to be infected by Bcc or *Aspergillus* species by sputum culture (*P* = 0.037, 0.011 respectively), with a trend towards increased infection with *P. aeruginosa* (*P* = 0.085).

### Individual sample and genus characteristics

**Figure 1:**
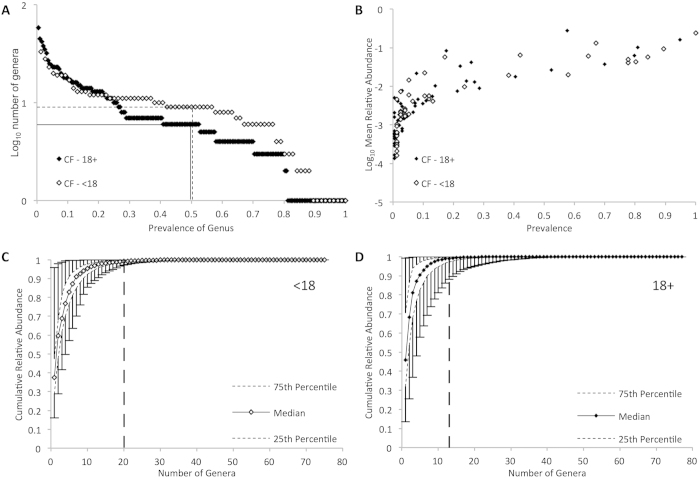
Genus level summary of bacterial population structure in cystic fibrosis (CF) in pediatric and adult sputum samples by 16S rRNA gene sequencing, after exclusion of operational taxonomic units that account for less than 0.005% sequence in the entire dataset. (**A**) Number of genera in pediatric (open diamonds) and adult (filled diamonds) specimens plotted by prevalence. Dashed (pediatric) and solid (adult) lines indicate core microbiota (genus present in >50% of samples). (**B**) Mean relative abundance of genera in pediatric (open diamonds) and adult (filled diamonds) specimens plotted by prevalence (**C** and **D**) Cumulative relative abundance of genera within a sputum sample of pediatric (**C**) and adult (**D**) CF patients plotted by number of genera. Error bars indicate range of values, and vertical dashed lines indicate the number of OTUs accounting for 99% of total sequence.

After quality and abundance filtering, 87 genera were identified across all samples. The size of the core microbiota of adult and pediatric samples, arbitrarily defined in our study as genera with a relative abundance of ≥1% of sequence in ≥50% of samples, were similar in both groups (Fig. 1A, Table 2). *Streptococcus, Prevotella, Rothia, Veillonella* and *Actinomyces* were core genera in both groups, while *Neisseria, Haemophilus* and *Gemella* were unique core genera in pediatric samples and *Pseudomonas* was unique to the adult core microbiota. In both groups, the genera with the greatest prevalence also had the greatest mean relative abundance (Fig. 1B).

A small subset of genera comprised most of the bacterial sequences detected in any sample (Fig. 1C and D, Table 3). The median number of genera required to account for half of the sequences in each sample was two in both groups, while a median of 20 and 13 genera accounted for 99% of the sequence in pediatric and adult patients, respectively (Table 3).

A dominant genus (the most abundant genus with at least twice the abundance of the second most abundant genus) was present in 45% of pediatric samples and 57% of adult samples. *Streptococcus, Haemophilus, Pseudomonas, Staphylococcus* and *Achromobacter* were dominant in multiple pediatric samples in descending order of frequency, while *Neisseria* and *Stenotrophomonas* were each dominant in one pediatric sample. *Pseudomonas, Burkholderia, Streptococcus, Haemophilus* and *Staphylococcus* were the dominant genus in descending order of frequency in adults (Table 2). The median relative abundance of the most abundant genus in each sample was 0.38 in pediatric samples (IQR 0.29-0.50) and 0.46 in adults (IQR 0.32-0.71), the median relative abundance of dominant genera was 0.64 (range 0.26-0.99).

*Rothia*, *Prevotella, Gemella, Actinomyces* and *Veillonella* were prevalent in both pediatric and adult samples, but were seldom or never the dominant genus. *Pseudomonas* was more prevalent in adults but tended to be dominant in both pediatric and adult samples in which it was present (Table 2). *Streptococcus*, *Burkholderia, Achromobacter, Stenotrophomonas* and *Haemophilus* were commonly the dominant genus in samples in which they were present. *Bordetella* accounted for 95% of the sequence in one adult specimen from a patient identified by culture as being chronically infected by *Bordetella avium*, but was not present in any other samples. In addition to having a high dominance proportion (the number of samples in which the genus was dominant divided by the total number of samples in which it was present with >1% relative abundance), *Pseudomonas* and *Burkholderia*, when dominant, accounted for a larger proportion of the community than *Streptococcus* in *Streptococcus*-dominant samples (72.8%, 78.2% and 47.1% of sequence in adult samples respectively, *P* < 0.0005). Only five genera (*Pseudomonas, Burkholderia, Stenotrophomonas, Haemophilus* and *Bordetella*) comprised more than 90% of any sample, and only in samples from adult patients.

### Within sample (alpha) diversity

**Figure 2:**
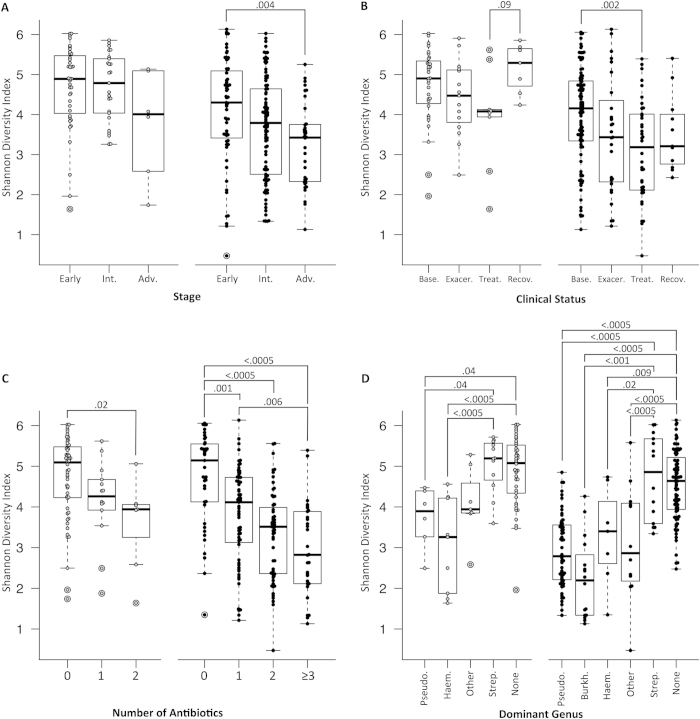
Within-sample (alpha) diversity of sputum specimens from CF patients. Boxplots represent minimum-25^th^ centile-median-75^th^ centile-maximum values and markers indicate individual patients. Open markers indicate pediatric patients and filled markers indicate adult patients. Alpha diversity was summarized using the Shannon diversity index, with diversity increasing on the y axis. Samples were grouped and compared by (**A**) disease stage (**B**) clinical status, (**C**) number of antibiotics being administered at the time of sample collection, and (**D**) dominant genus. *P*-values represent the results of t-tests between groups, within pediatric and adult cohorts. Stage, clinical status, treatment groups and dominant genus are defined in the Methods section. Abbreviations: Int.= intermediate, Adv.= advanced, Base.= baseline, Exacer. = exacerbation, Recov. = recovery, Pseudo. = *Pseudomonas,* Haem. = *Haemophilus,* Strep. = *Streptococcus*, Burk. = *Burkholderia*, None = No dominant genus.

Advanced disease stage was associated with lower diversity in adult patients (*P* = 0.004, Fig. 2A). This difference was not statistically significant in pediatric patients; however, only 6 pediatric patients had advanced disease. Baseline samples were more diverse than treatment samples for adults (*P* = 0.002, Fig. 2B). There was a stepwise, statistically significant decrease in diversity associated with increased number of antibiotics prescribed in both cohorts (Fig. 2C). *Streptococcus* dominance was associated with higher community diversity in both datasets (Fig. 2D).

### Between sample (beta) diversity

**Figure 3:**
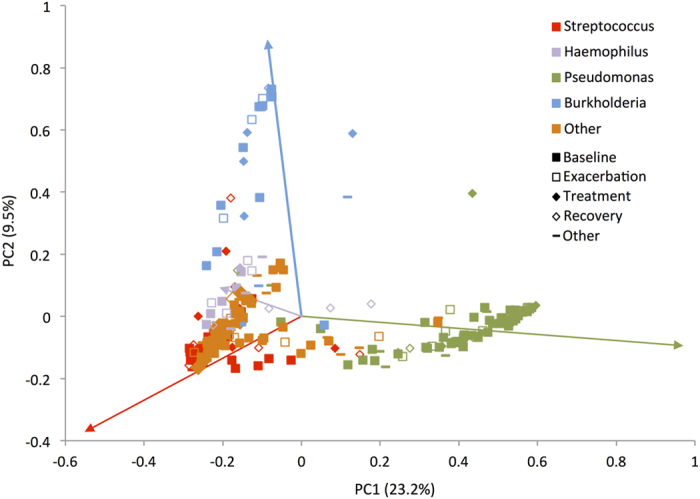
Between-sample (beta) diversity of sputum specimens from CF patients. Bray-Curtis dissimilarity principal coordinates analysis (PCoA) was used to generate ordination of beta-diversity in two dimensions. Principal coordinates 1 and 2 (PC1 and PC2) explain 23.2% and 9.5% of the variance in Bray-Curtis dissimilarity respectively (x and y axes). Increasing distance in two-dimensional space represents increasingly dissimilar community structures. The marker style indicates samples were obtained from patients at clinical baseline, exacerbation, treatment, recovery or other as defined in the Methods section. Samples are colored according to most abundant genus. Colored arrows in the plot indicate Pearson correlation coefficients between genus relative abundance and each principal coordinate, with the line colors corresponding to the genus color in the legend.

**Figure 4:**
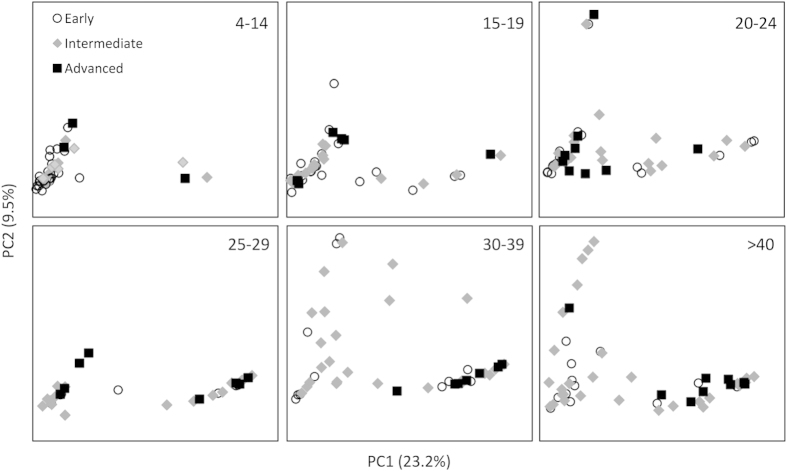
PCoA by Bray-Curtis dissimilarity was plotted by age group as indicated in the upper right corner of each panel. Markers represent individual patients and marker style indicates disease stage at the time of sample collection. Principal coordinates 1 and 2 correlate with relative abundance of *Pseudomonas* and *Burkholderia*, respectively as in Fig. 3.

We assessed inter-sample variability in community structure (beta diversity) using unsupervised principal coordinates analysis (PCoA) of Bray-Curtis dissimilarity (Fig. 3). The relative abundance of *Pseudomonas, Burkholderia*, *Streptococcus* and *Haemophilus* largely drove ordination (Fig. 3). Samples did not cluster by clinical status at the time of sample acquisition (baseline, exacerbation, treatment, recovery or other). Notably, Bray-Curtis ordination was influenced by disease stage differently across the age groups, with clustering based on disease stage becoming more apparent after age 30 (Fig. 4). This analysis did not reveal any grouping of bacterial community structures by gender, CFTR genotype, pancreatic status, FEV_1_, FVC or BMI.

### Age group and disease status associated bacterial community changes

**Figure 5:**
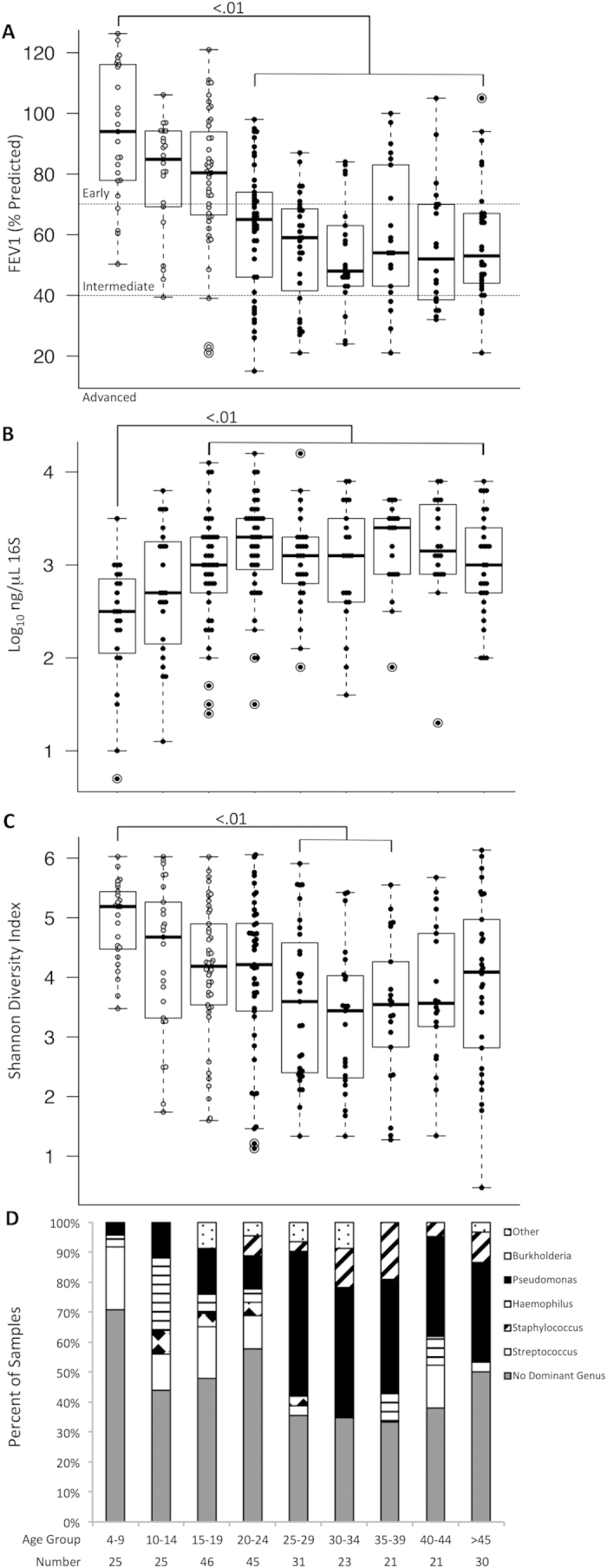
(**A**) Age dependent changes in lung function (FEV_1_% predicted), (**B**) bacterial load (ng/μL 16S rRNA), (**C**) alpha diversity (Shannon diversity index) and (**D**) dominant genus (defined in Methods) demonstrate concordant changes in lung function, diversity and increased prevalence of *Pseudomonas* and *Burkholderia* with increasing age. Chi-squared significance for panel D is P < 0.0005.

Patients were stratified into age groups and compared based on lung function, community diversity and dominant genera (Fig. 5). As expected, FEV_1_ was lower in older age groups, with the lowest median FEV_1_ in patients aged 30-34 years (Fig. 5A). Bacterial density (log_10_ ng/μL of 16S rRNA gene by quantitative PCR) was greatest at ages 20-24 (Fig. 5B). Diversity was also lower in older age groups, and differences in community diversity paralleled lung function across age groups (diversity nadir 30-34 years, Fig. 5C). In addition, the lower lung function and diversity observed in older age groups coincided with a higher proportion of samples dominated by *Pseudomonas* or *Burkholderia* and a decreased proportion of *Streptococcus* dominance and samples without a dominant genus (Fig. 5D). If dichotomized at age 25, *Streptococcus* dominance and no dominant genus account for 69% of the samples from younger patients, but only 43% of samples from older patients, whereas *Pseudomonas* and *Burkholderia* dominant samples comprise only 14% of samples from patients under 25 years but 49% of cases from patients 25 years or older (Chi-squared of dominant taxa in <25 or ≥25 year old patients, P < 0.0005).

### Predictors of FEV_1_ by multivariate linear regression

Given the observed correlation of bacterial diversity, density and dominant taxa for lung function across age groups, we performed multivariate linear regression to ascertain which features of the lung microbiota independently predict lung function while controlling for the influence of host factors. Factors with known significance and potential confounders present in our dataset were entered into the multivariate model prior to sequentially testing diversity (SDI), bacterial density (log_10_ 16s rRNA gene by quantitative PCR) and dominant taxa based on the strategy outlined in the methods section. The final model included age, BMI z-score, pancreatic insufficiency, SDI and *Pseudomonas* dominance (adjusted *R*^*2*^ = 0.315, df = 5, F = 22.9, P < 0.0005, Table 4). Increasing age, pancreatic insufficiency, increasing number of antibiotics and *Pseudomonas* dominance negatively correlated with lung function, while increasing BMI and diversity positively correlated with lung function in our model.

## Discussion

We describe the bacterial community structure of sputum samples from 269 cystic fibrosis patients across a broad range of age, disease stage, clinical status and treatment using a culture-independent method. This is the largest cohort of CF patients spanning both adult and pediatric populations for whom sputum has been analysed by 16S rRNA gene sequencing yet published, and includes a large number of patients younger than 18 years of age. We observed several clear features of bacterial community structure in CF sputum and their relationship with disease states.

Firstly, the airways of CF patients share only a small number of common taxa, but otherwise demonstrate a high degree of inter-individual variability in community structure, composition and diversity, confirming the results of smaller studies^5^,^6^,^7^. In pediatric and adult CF patients, the shared core microbiome was small, and consisted largely of genera not considered to be traditional CF pathogens, namely *Streptococcus, Rothia, Veillonella* and *Actinomyces*. *Pseudomonas*, a prototypic CF pathogen, was a member of only the adult core microbiota. A small number of taxa accounted for the bulk of bacterial sequences present in any sample, and approximately half of all patients had a bacterial community dominated by a single genus.

Secondly, the observed taxa in the CF lung vary in their relative abundance and dominance of the bacterial community. Only six genera had a dominance proportion >0.10 and a maximum relative abundance >0.70: *Pseudomonas, Burkholderia, Stenotrophomonas, Achromobacter, Haemophilus* and *Bordetella*. Of the remaining genera, only *Staphylococcus, Streptococcus* and *Bifidobacterium* were observed at a relative abundance of at least 0.50 in one or more samples.

In addition, whereas within sample diversity correlated with several patient, treatment and microbial factors, between patient diversity was largely driven by the age-associated relative abundance of *Pseudomonas* and *Burkholderia*. Age, disease stage, treatment and dominant genus were found to significantly correlate with differences in alpha diversity, confirming the observations of previous published reports of smaller datasets^8^,^11^,^12^,^13^,^16^,^17^,^18^,^19^,^20^. The largest differences between patient communities noted in our study were the greater prevalence and relative abundance of *Pseudomonas* and *Burkholderia* in older age groups. As patients with CF age, their risk of acquisition of these organisms increases^17^,^21^, and we observed a stepwise progression in the prevalence and relative abundance of these two important genera with increasing age. A notable increase in *Pseudomonas* and *Burkholderia* dominance was observed in patients aged 25 and older, which coincided with the lowest lung function and sample diversity observed in any age group. Beyond this age, there are few differences in overall community structure, diversity or lung function in surviving adult patients, and it is possible that the establishment of these CF associated pathogens is largely complete by age 20-25. These findings corroborate the findings of smaller cross-sectional studies of CF microbiota over a large age range, including the ‘plateau’ of community and functional change at approximately 25 years of age^17^,^22^.

This study also demonstrated that sample diversity was positively correlated with lung function after controlling for host factors. Multivariate regression of clinical and bacterial features for prediction of lung function revealed that age, BMI z-score, pancreatic insufficiency, diversity and *Pseudomonas* dominance independently predict lung function. The observation that diversity positively correlates with lung function agrees with other studies in CF and non-CF bronchiectasis^8^,^9^,^23^. Our and previously published analyses do not address causality, however, and it remains uncertain whether the relationship between disease stage and diversity is causal.

This study has important caveats. The prevalence of infection with *Pseudomonas* in this study was lower than published registry data for both pediatric and adult patients^24^. Similarly, none of the included specimens were culture-positive for *Mycobacterium*, despite recently reported prevalence rates of nearly 10%^25^. However, prevalence of all species by culture in our study was determined for the study sample only, not for historical samples from each patient, and thus is unsurprisingly lower than published reports which generally define infection as culture positivity within a time range, rather than at a single timepoint.

Adequacy of sampling in CF microbiome studies is difficult to conclusively ascertain and is a challenge in similar studies. Culture-independent analysis by 16S rRNA gene sequencing is not uniformly sensitive to the detection of all bacterial taxa, such as *Staphylococcus*, in the absence of specific sample processing procedures^26^. Our study was not designed to systematically compare within-sample relationships between culture and 16S rRNA sequence data. As such, our analysis of *Staphylococcal* relative abundance should be interpreted cautiously.

Since our cohort did not include paired specimens from the same patient, this analysis was not designed to identify differences in bacterial community characteristics occurring at times of different disease activity (such as during an exacerbation). Given the highly variable bacterial community structure in CF which we and others^5^,^6^,^7^ have observed, between-patient differences are likely to obscure important within-patient trends occurring during exacerbations.

Furthermore, our analysis was not designed to assess the impact that rare or very low abundance taxa (<1% relative abundance) may have on the biology of the CF airway, but rather to assess trends in community structure over a broad range of ages and disease stage. Very low abundance and rare taxa may be critical determinants of pathological processes in the CF lung. Our observations highlight the heterogeneity between patients and confirm the need for longitudinal analysis in individual patients and groups of patients to identify factors associated with disease exacerbations, disease progression and response to treatment. Our method resolved community composition to the genus level only. Significant within-genus or intra-species variability in community composition may have important effects on community behavior and disease pathogenesis and has the potential to explain cross-sectional variability in disease status that is not apparent at the genus level.

Despite these caveats, our data agree with prior reports of CF microbiota, and extend these observations to a broader age range of patients, particularly those less than 18 years of age, a group that is not well represented in previously published reports. Our principal findings agree with and extend those of smaller studies of mostly adult patients showing high inter-individual variability in community structure^5^,^6^,^7^, a common core microbiota^9^, age associated changes in community composition^16^,^17^, and disease stage associated decreases in diversity^8^,^11^,^12^,^13^,^16^,^17^,^18^,^19^,^20^.

Longitudinal studies identifying microbial and host risk factors for infection with patho-adapted taxa such as *Pseudomonas* and *Burkholderia,* and mechanistic studies elucidating the interactions of these taxa with other colonizing organisms, as well as exploration of possible causal relationships between community diversity and disease manifestations would be illuminating.

## Methods

### Study enrollment, sample and data collection

This study received approval from the research ethics boards of both St. Michael’s Hospital and the Hospital for Sick Children in Toronto, Canada. Patients provided informed consent and were enrolled prospectively at The Hospital for Sick Children and St. Michael’s Hospital between 2010 and 2013. Height, weight, forced expiratory volume in one second (FEV_1_), forced vital capacity (FVC), age, sex, disease stage, antimicrobial therapy, clinical status, cystic fibrosis transmembrane conductance regulator (CFTR) mutation, pancreatic status, and visit outcome were recorded for the day of sample collection. Expectorated sputa were collected for all patients - no oropharyngeal samples were used. Pediatric samples were split into aliquots, one for culture and one for nucleic acid extraction, while adults provided two samples in parallel at each clinic visit. Samples for sequencing were treated with sputolysin and frozen for subsequent processing. Quantitation of 16S rRNA gene copy number was performed by quantitative PCR based on published protocols^27^.

### Si-Seq 16S rRNA gene analysis and informatics

Sequencing was performed at the Centre for the Analysis of Genome Evolution and Function (CAGEF) at the University of Toronto as previously described using an Illumina GAIIx^28^. An in-house Java script was used to prepare the sequence for downstream analysis using MACQIIME^29^. Chimeric reads were removed using reference-based chimera detection with USEARCH^30^. Operational taxonomic units (OTUs) were picked against a SI-Seq structured reference set using a similarity of 0.87 (corresponding to the genus level with the SI-Seq method), with premature termination turned off. Taxonomy was assigned using an RDP structured dataset using UCLUST^30^ with --max_accepts set to 0 and similarity set to 0.97, (empirically determined to produce the highest consistency identification at the genus level using *Pseudomonas aeruginosa* controls)^28^. Unassigned OTUs were identified with BLASTN^31^. OTUs representing less than 0.005% of overall sequence were removed. At this stage, the median number of sequences/sample was 67,812 (IQR 42,835-87,773). For calculation of Shannon diversity index (SDI), samples were rarefied to 1500 sequences to account for the effects of sequencing depth on diversity, with a median Good’s coverage of 0.972 (IQR 0.955-0.979) at that sampling depth. Diversity and principal coordinates analyses were performed using MACQIIME with default settings.

### Statistical analyses

Statistical analyses were performed using SPSS version 22 (IBM). Means were compared using T-tests or ANOVA with Bonferroni correction as needed. Chi-squared tests were used to test proportions. A multivariate linear regression model predicting FEV_1_ (% predicted) was built by entering all variables, then using backwards removal to identify variables in the final model.

### Definitions

Disease stage, pancreatic insufficiency, FEV_1_, and FVC were defined as previously reported^32^,^33^. BMI z score was calculated using the method described in Flegal *et al.*^34^ using age-specific parameter values from the Centers for Disease Control ( www.cdc.gov/growthcharts/percentile_data_files.htm). For adults over 20 years old, the age values for BMI z score were calculated based on an age of 20^35^. Antibiotic number equaled the number of antibacterials of any type being taken on the day of the visit (including inhaled antibiotics). Clinical status was defined as ‘baseline’, ‘exacerbation’, ‘treatment’ or ‘recovery’ using the criteria of Zhao *et al.*^11^. Samples not meeting these criteria were categorized as ‘other’ and included patients receiving new prescriptions for steroids, antifungals or antibiotics for an indication other than a pulmonary exacerbation (e.g. an extra-pulmonary infection). ‘Dominant genus’ was defined as any genus whose relative abundance was at least twice that of the next most abundant genus^10^ or if no such genus was present, ‘no dominant genus’.

All methods were performed in accordance with the approved guidelines.

## Author Contributions

B.C., D.M.H. and D.S.G. drafted the manuscript. B.C. created all figures and tables. D.E.T., S.D., S.T.C., V.B., Y.C.W.Y., V.J.W. and Y.Z. enrolled patients, collected clinical data and samples, and processed samples for sequencing. P.W.W., A.S., Y.G., J.D.C., S.T.C. and B.C. sequenced samples and processed sequence data. B.C., J.D.C., A.S., Y.G., V.J.W., D.M.H. and D.S.G. analyzed and interpreted the data. B.C., Y.C.W.Y., V.J.W., D.M.H., D.S.G. conceived the study design. All authors reviewed the manuscript.

## Additional Information

**How to cite this article**: Coburn, B. *et al.* Lung microbiota across age and disease stage in cystic fibrosis. *Sci. Rep.* **5**, 10241; doi: 10.1038/srep10241 (2015).

## Figures and Tables

**Table 1:** Study participant summary by age category.

	**<18**	**18+**	**P-value**
N	76	193	-
Median Age (Range)	12 (4-17)	29 (18-64)	-
Male (%)	33 (43.4)	112 (59.2)	0.041
			
CFTR Genotype			
dF508/dF508	40 (52.6)	87 (45.8)	
dF508/other	23 (30.3)	69 (36.3)	ns
other/other	13 (17.1)	34 (17.9)	
Median BMI Z-score (<18), BMI (18+) (Range)	−0.6 (−3-1.4)*	22.2 (15.3-38.3)	-
Pancreatic insufficiency (%)	71 (93.4)	129 (72.1)*	<0.001
			
Disease stage			
Early	39 (55.7)^*^	59 (31.4)*	
Intermediate	25 (35.7)*	94 (50.0)*	<0.001
Advanced	6 (8.6)*	35 (18.6)*	
FEV_1_% predicted (median, range)	73 (26.6-117)*	60.5 (15-111)*	<0.001
			
Clinical status at sputum collection			
Baseline	32 (42.1)	100 (51.8)	
Exacerbation	15 (19.7)	27 (13.9)	
Treatment	9 (11.8)	38 (19.7)	<0.001
Recovery	7 (9.2)	11 (5.7)	
Other	13 (17.1)	17 (8.8)	
			
Sputum culture positivity at study visit			
*Pseudomonas aeruginosa*	19 (25.0)	71 (37.2)	0.085
*Burkholderia cepacia* complex	4 (5.3)	28 (14.7)	0.037
*Stenotrophomonas maltophilia*	8 (10.5)	19 (9.9)	ns
*Mycobacterium species*	0	0	-
*Aspergillus* species	13 (17.1)	64 (33.5)	0.011

ns - not significant

^*^Indicates missing data

**Table 2:** Dominance proportion of selected genera across specimens.

**Genus**	Present* **N (% of all samples)**	Most abundant N (% of all samples)	Dominant^t^ **N (% of all samples)**	Dominance proportion§	Maximum Relative Abundance
<18 years					
Streptococcus	76 (100.0)	34 (44.7)	12 (15.8)	0.16	0.58
Rothia	67 (88.1)	6 (7.9)	0	0	0.41
Prevotella	64 (84.2)	3 (3.9)	0	0	0.43
Actinomyces	61 (80.2)	1 (1.3)	0	0	0.20
Veillonella	59 (77.6)	0	0	0	0.18
Gemella	58 (76.3)	0	0	0	0.17
Haemophilus	51 (67.1)	13 (17.1)	9 (11.8)	0.18	0.81
Neisseria	49 (64.5)	1 (1.3)	1 (1.3)	0.02	0.29
Staphylococcus	32 (42.1)	6 (7.9)	3 (3.9)	0.09	0.96
Porphyromonas	29 (38.2)	0	0	0	0.18
Fusobacterium	18 (23.7)	0	0	0	0.23
Pseudomonas	13 (17.1)	6 (7.9)	6 (7.9)	0.46	0.86
Solobacterium	9 (11.8)	0	0	0	0.12
Leptotrichia	8 (10.5)	0	0	0	0.06
Stenotrophomonas	8 (10.5)	1 (1.3)	1 (1.3)	0.13	0.57
Oribacterium	5 (6.6)	0	0	0	0.04
Achromobacter	4 (5.3)	2 (2.6)	2 (2.6)	0.50	0.52
Campylobacter	2 (2.6)	0	0	0	0.02
Burkholderia	2 (2.6)	1 (1.3)	0	0	0.51
Lactobacillus	1 (1.3)	0	0	0	0.04
Bifidobacterium	1 (1.3)	0	0	0	0.02
Scardovia	1 (1.3)	1 (1.3)	0	0	0.33
					
18+ years					
Streptococcus	183 (94.8)	38 (19.7)	14 (7.3)	0.08	0.78
Prevotella	156 (80.8)	24 (12.4)	2 (1)	0.01	0.39
Rothia	154 (79.8)	2 (1)	0	0	0.35
Veillonella	135 (69.9)	0	0	0	0.22
Pseudomonas	111 (57.5)	71 (36.8)	61 (31.6)	0.55	0.99
Actinomyces	101 (52.3)	1 (0.5)	0	0	0.21
Gemella	78 (40.4)	1 (0.5)	0	0	0.16
Porphyromonas	52 (26.9)	0	0	0	0.17
Haemophilus	50 (25.9)	9 (4.7)	7 (3.6)	0.14	0.90
Fusobacterium	47 (24.4)	2 (1)	0	0	0.42
Staphylococcus	43 (22.3)	9 (4.7)	4 (2.1)	0.09	0.64
Burkholderia	34 (17.6)	22 (11.4)	16 (8.3)	0.47	0.97
Neisseria	27 (14.0)	1 (0.5)	0	0	0.21
Oribacterium	25 (13.0)	0	0	0	0.11
Leptotrichia	16 (8.3)	2 (1)	0	0	0.20
Stenotrophomonas	15 (7.8)	5 (2.6)	3 (1.6)	0.20	0.95
Solobacterium	14 (7.3)	0	0	0	0.19
Campylobacter	13 (6.7)	0	0	0	0.03
Achromobacter	6 (3.1)	2 (1)	1 (0.5)	0.17	0.73
Lactobacillus	6 (3.1)	0	0	0	0.20
Bifidobacterium	5 (2.6)	1 (0.5)	1 (0.5)	0.20	0.58
Inquilinus	3 (1.6)	1 (0.5)	0	0	0.32
Bordetella	1 (0.5)	1 (0.5)	1 (0.5)	1.00	0.95
Proteus	1 (0.5)	1 (0.5)	0	0	0.30

^*^Specimen >0.01 relative abundance

^t^Dominance = Genus is most abundant AND > 2x the relative abundance of next most abundant genus

^§^=(Number of samples in which genus is dominant)/(Number in which it is present with RA >0.01)

**Table 3:** Relative sequence abundance by number of genera in each patient.

	**<18 (median, range)**	**18+ (median, range)**
Number of genera >0.01 relative abundance	10 (2-15)	8 (1-17)
OTU_50_	2 (1-5)	2 (1-5)
OTU_75_	4 (1-8)	3 (1-9)
OTU_90_	7 (1-13)	5 (1-15)
OTU_99_	20 (4-28)	13 (1-35)

OTU_50_: number of OTUs that represent 50% of the overall sequence (per patient)

OTU_75_: number of OTUs that represent 75% of the overall sequence (per patient)

OTU_90_: number of OTUs that represent 90% of the overall sequence (per patient)

OTU_99_: number of OTUs that represent 99% of the overall sequence (per patient)

**Table 4:** Multivariate regression model for predicting lung function (FEV_1_ % predicted).

	Initial Model*	**Final Model**^t^		
Variable	**β (95% CI, lower, upper bound)**	**p-value**	**β (95% CI, lower, upper bound)**	**p-value**
Constant	84.9 (65.8,104.2)	<0.0005	80.6 (66.5,94.6)	<0.0005
Age (years)	−0.6 (−0.8,−0.4)	<0.0005	−0.6 (−0.8,−0.4)	<0.0005
Body Mass Index	7.5 (5,10)	<0.0005	7.3 (4.9,9.8)	<0.0005
Pancreatic Insufficiency	−9.5 (−17,−2.1)	0.012	−8.8 (−15.4,−2.2)	0.009
Antibiotic number	−1.8 (−4.1,0.5)	0.132	-	-
Male sex	2.8 (−2.4,8)	0.291	-	-
F508 homozygous	0.8 (−4.5,6.1)	0.768	-	-
Diversity (SDI)	2.2 (−0.4,4.8)	0.098	2.5 (0.5,4.6)	0.016
Log_10_ 16S (ng/μL)	−1.2 (−5.1,2.6)	0.532	-	-
*Dominant genus*				
Pseudomonas	1.2 (−11,13.5)	0.107	−7.6 (−13.7,−1.5)	0.015
Burkholderia	−0.5 (−8.9,7.9)	0.844	-	-
Streptococcus	7.8 (−3.7,19.3)	0.904	-	-
Haemophilus	−3.9 (−13.4,5.7)	0.182	-	-
Other	84.9 (65.8,104.2)	0.426	-	-

^*^Preliminary model = statistics at the time of variable entry as described in materials and methods.

^t^Final Model = statistics at the time of finalization of the multivariate model with all final variables included.
